# Apis—an Involuntary Proving

**Published:** 1893-07

**Authors:** 


### APIS—AN INVOLUNTARY PROVING.

A bee stung me on the helix of my left ear one hot June day. I give the symptoms in the order of their sequence, so far as the brain remained clear enough to note them: 1. Sensation as though a large stick like a broom-handle were thrust through my head from left to right. 2. Swelling of the entire person. 3. Eruption like a nettle-rash, covering the entire surface, even the palms of the hands and the soles of the feet. 4. Severe nervous chill, with chattering of teeth and shivering, but without sensation of cold. 5. Complete suppression of urine, with pain in the kidneys and bladder. 6. Dull pain in the entire head, with sensation of weariness of the brain, and a stupid condition, with inability to note symptoms further. (At this juncture my husband administered a gill of Holland gin. I had taken a sponge bath of ammonia and water, and was placed in bed.) *Secondary Symptoms.*—After a restless sleep noted the following conditions: 1. Retention of urine followed after a few hours by a scanty discharge of red, hot urine. Pain and soreness in the region of the kidneys, bladder, and ovaries. 2. Eruption disappeared, leaving the skin white, waxy, and a condition of general œdema. 3. Extreme sensitiveness to touch and soreness on deep pressure. 4. Brain symptoms slowly relieved. 5. Soreness of muscles and stiffness of joints like rheumatism. At the end of a week was restored to normal condition.—Julia C. Jump, M. D., Oberlin, O.—*North American Journal of Homœopathy,* Vol. VIII, No. 5, May, 1893.

